# A case report and literature review of steroid-refractory pancreatitis induced by cadonilimab in patient with lung squamous cell carcinoma

**DOI:** 10.1097/MD.0000000000047354

**Published:** 2026-01-23

**Authors:** Rui Qiu, Gao-feng Zheng, Min Zhong, Jin-lan Li

**Affiliations:** a Department of Pharmacy, The People’s Hospital of Jianyang City, Jianyang, Sichuan, China; b Department of Oncology, The People’s Hospital of Jianyang City, Jianyang, Sichuan, China; c Department of Pharmacy, Hunan Cancer Hospital/The Affiliated Cancer Hospital of Xiangya School of Medicine, Central South University, Changsha, Hunan Province, China.

**Keywords:** cadonilimab, case report, mycophenolic acid, non-small-cell lung cancer, pancreatitis

## Abstract

**Rationale:**

Cadonilimab-induced pancreatitis is an uncommon occurrence. This article seeks to present a case study of pancreatitis attributed to cadonilimab, detailing its onset and therapeutic approach. Additionally, it examines the characteristics, underlying mechanisms, and management strategies of cadonilimab-induced pancreatitis through a comprehensive literature review.

**Patient concerns:**

A 44-year-old male patient was diagnosed with squamous cell carcinoma of the right lower lung 21 months prior. Following multiple lines of antitumor therapy, he commenced treatment with cadonilimab plus anlotinib. Twenty-two days subsequent to the initiation of this combination therapy, the patient experienced intermittent abdominal pain, and laboratory tests revealed elevated amylase levels. Despite these findings, the patient was monitored and continued the immunotherapy and targeted treatment regimen. Sixty-two days later, follow-up laboratory tests demonstrated further elevation in amylase levels, and magnetic resonance imaging indicated pancreatic enlargement.

**Diagnoses:**

Grade 3 immune-related pancreatitis.

**Interventions:**

The patient was administered high-dose methylprednisolone therapy.

**Outcomes:**

During the course of this treatment, the patient exhibited exacerbated abdominal pain and was subsequently diagnosed with steroid-refractory pancreatitis. Consequently, a combination therapy including mycophenolate mofetil was initiated, resulting in a reduction of serum amylase levels and alleviation of abdominal pain.

**Lessons:**

Cadonilimab-related pancreatic injury is uncommon and typically presents as asymptomatic hyperlipasemia. Immune-related pancreatitis is an infrequent adverse event. Clinicians and clinical pharmacists are advised to remain vigilant regarding this condition. Additionally, this case offers valuable insights into the management of steroid-refractory pancreatitis.

## 1. Introduction

Cadonilimab is a bispecific antibody that targets human programmed cell death 1 (PD-1) and cytotoxic T lymphocyte antigen 4 (CTLA-4), thereby enhancing tumor-specific T-cell immunity by inhibiting the immune-suppressive responses associated with the PD-1 and CTLA-4 signaling pathways. This therapeutic agent received approval and was introduced to the Chinese market on June 29, 2022.^[1]^ Empirical evidence indicates that cadonilimab exhibits promising efficacy and manageable safety profiles across various tumor types.^[2]^ Common adverse effects associated with cadonilimab include rash, anemia, hypothyroidism, and elevated levels of aspartate aminotransferase and alanine aminotransferase. To date, there have been no documented cases of immune-related pancreatitis attributable to this drug. This study presents an analysis of a case involving steroid-refractory pancreatitis in a patient with advanced squamous cell lung cancer undergoing treatment with cadonilimab, with the objective of providing insights for the rational clinical application of this medication.

## 2. Case presentation

### 2.1. Clinical materials

**Figure 1.**
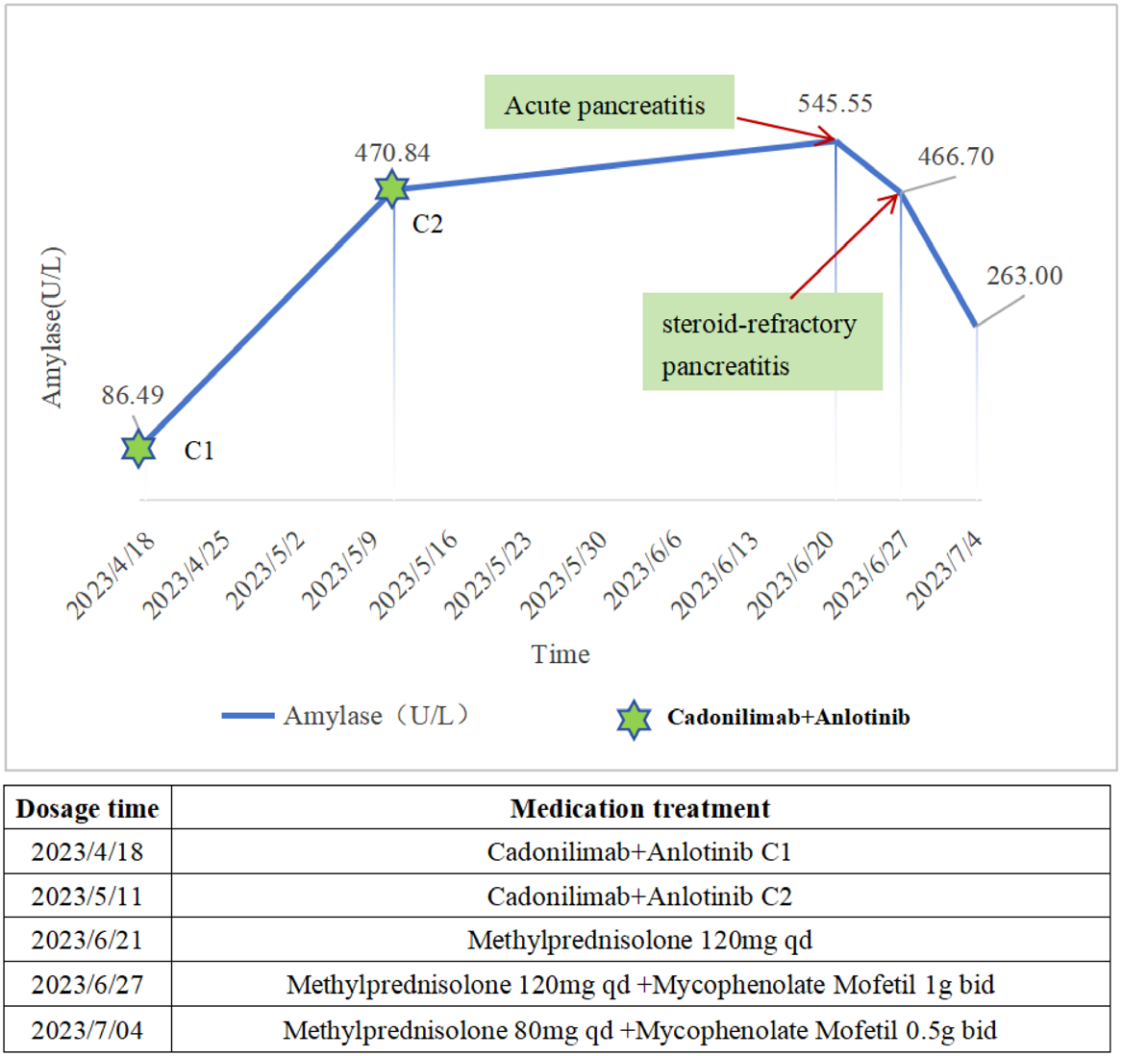
Temporal evolution of immune-related pancreatitis. C1: first cycle of cadonilimab plus anlotinib; C2: second cycle of cadonilimab plus anlotinib. Acute pancreatitis: the patient had persistent abdominal pain and continuously elevated amylase levels; MRI of the pancreas revealed enlargement and signal abnormalities in the body and tail of the pancreas. Steroid-refractory pancreatitis: the patient’s amylase level initially decreased after corticosteroid therapy but subsequently rose again, accompanied by worsening abdominal pain. MRI = magnetic resonance imaging.

A 44-year-old male patient was diagnosed with centrally located squamous cell carcinoma of the right lower lung, classified as stage cT_4_N_3_M_1c_, IV_B_, with metastases to the bilateral supraclavicular, right axillary, and retroperitoneal lymph nodes. Twenty-one months prior, the patient had been diagnosed with squamous cell carcinoma of the right lower lung at a different medical facility and had undergone 4 cycles of chemotherapy with pembrolizumab in combination with the paclitaxel and carboplatin regimen, resulting in a partial remission. Eighteen months ago, due to the inoperability of the tumor, the patient received chemotherapy with nab-paclitaxel, sintilimab, and recombinant human endostatin at a local hospital. Ten months ago, the regimen was modified to 3 cycles of sintilimab plus anlotinib; however, the disease exhibited progression. Eight months ago, the patient underwent 30 sessions of thoracic radiotherapy, but the disease continued to progress. Four months prior, the patient was enrolled in a clinical trial and received 2 cycles of SHR-A1921 treatment, during which the disease continued to progress. Two months ago, following the recommendation of a multidisciplinary consultation and the patient’s informed consent, a 21-day regimen was initiated: cadonilimab 750 mg intravenously on day 1 plus anlotinib 8 mg orally once daily from days 1 to 14. Forty days ago, the patient returned to the hospital for the second cycle of combined immunotherapy and targeted therapy. The patient reported occasional but tolerable abdominal pain. The serum amylase level was measured at 470.84 U/L, exceeding the reference range of 35.00 to 135.00 U/L. Following discussions between the clinical physician and the patient, they decided to monitor the condition while proceeding with the second cycle of combined immunotherapy and targeted therapy. The patient was admitted this time for the third cycle of antitumor treatment. Temporal evolution of immune-related pancreatitis is shown in Figure 1.

According to “Measures for the Ethical Review of Biomedical Research Involving Human Beings” (Order No. 11 of the National Health Commission, 2016) in China and the “Declaration of Helsinki,” informed consent for publication was waived based on the retrospective noninterventional design and anonymous management of the patients’ data. Ethical approval for publication of this case report was obtained from Hunan Cancer Hospital ethics committee (approval number: 2025 scientific research simplified procedure review number 65).

### 2.2. Physical examination at admission

Upon admission, the patient’s physical examination revealed a body temperature of 36.9°C, a heart rate of 107 beats/min, a respiratory rate of 20 breaths/min, and a blood pressure of 131/89 mm Hg. The cardiac silhouette appeared normal in size, with regular pulsations and rhythm, and no murmurs were detected. The thorax was symmetrical and free of deformities, with no tenderness noted over the sternum. Respiratory movements were symmetrical and unobstructed bilaterally, and tactile fremitus was normal. Percussion of the thorax produced clear sounds, although breath sounds were diminished in the right lower lung field; the remaining lung fields exhibited clear breath sounds without any dry or wet rales. The abdomen was flat, with no visible veins on the abdominal wall and no observable gastric or intestinal patterns or peristaltic waves. The abdomen was soft, with no tenderness, rebound tenderness, or muscle rigidity. There was no evidence of shifting dullness or succussion splash, and no palpable masses were detected. The liver and spleen were not palpable below the costal margin, and Murphy’s sign was negative.

### 2.3. Laboratory examinations

**Figure 2.**
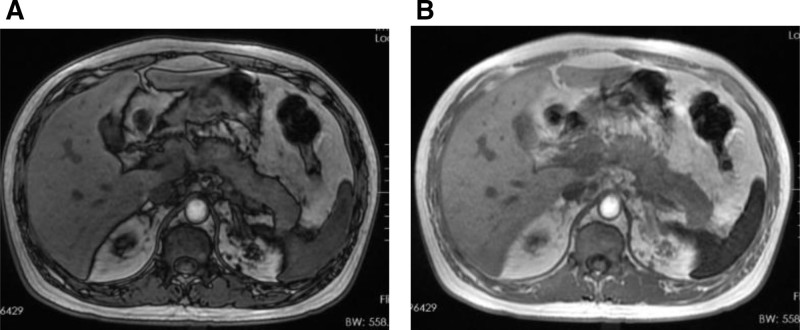
Patient’s pancreatic MRI. (A, B) The body and tail of the pancreas are mildly enlarged, exhibiting slightly prolonged T1 and T2 signals. On diffusion-weighted imaging (DWI), it shows slightly elevated signals. The enhancement is slightly nonuniform on the contrast-enhanced scan. DWI = diffusion-weighted imaging, MRI = magnetic resonance imaging.

Laboratory investigations revealed an elevated serum amylase level of 545.55 U/L. A contrast-enhanced thin-section magnetic resonance imaging (MRI) of the pancreas indicated mild swelling and abnormal signal intensities in the body and tail of the pancreas, accompanied by thickening of the left perirenal fascia, findings that are suggestive of acute pancreatitis (refer to Fig. 2).

### 2.4. Diagnostic assessment

Following admission, the patient persistently reported intermittent abdominal pain. Subsequent diagnostic evaluations revealed elevated serum amylase levels, and MRI indicated acute pancreatitis in the absence of gallstones. Laboratory analyses demonstrated a triglyceride concentration of 2.41 mmol/L (reference range: 0.40–1.86 mmol/L), which was lower than the triglyceride level recorded prior to the initiation of cadonilimab treatment (2.64 mmol/L). The patient denied any family history, and reported no history of alcohol abuse, overeating, or autoimmune disorders. He had previously received thoracic radiotherapy, which is associated with pneumonitis and thyroiditis^[3]^ but lacks evidence-based links to pancreatitis. Although abdominal irradiation can cause chronic pancreatitis,^[4]^ this patient had no history of abdominal radiation, excluding radiotherapy as a contributory factor. Regarding whether elevated triglycerides were related to the current episode of pancreatitis, the literature has not established a definitive threshold for hypertriglyceridemic pancreatitis; however, a level > 1000 mg/dL (11.3 mmol/L) is generally considered the pathogenic cutoff.^[5]^ At the time of the pancreatitis attack, this patient’s triglyceride level was 2.41 mmol/L, which was even lower than the pretreatment value, thereby excluding elevated triglycerides as a synergistic cause of this episode of pancreatitis.

The patient was diagnosed with immune-related pancreatitis 62 days following the initial administration of cadonilimab in conjunction with anlotinib. The severity of the condition, as per common terminology criteria for adverse even (CTCAE) version 5.0,^[6]^ was confirmed to be grade 3. Utilizing the Naranjo Adverse Drug Reaction Probability Scale,^[7]^ the causality assessment yielded a score of 5 for cadonilimab, suggesting a probable association with pancreatitis, while anlotinib received a score of 2, indicating a possible association. Anlotinib is a multi-target receptor tyrosine kinase inhibitor. As of October 2025, no case reports of anlotinib monotherapy-induced pancreatitis have been identified in CNKI, Wanfang, VIP, or PubMed. Clinical trials^[8,9]^ show that anlotinib monotherapy is accompanied by lipase elevation in 5.67% to 22.58% of patients and amylase elevation in 12.90%. Although the amylase rise observed in this case might be related to anlotinib, the fact that the patient continued anlotinib without recurrence of immune-mediated pancreatitis after discontinuation of cadonilimab markedly weakens the likelihood that anlotinib was the causative agent. Taken together, the evidence supports the conclusion that the episode of immune-related pancreatitis was “probable” induced by cadonilimab.

### 2.5. Therapeutic intervention

Initial treatment: cadonilimab was promptly discontinued, and the patient was administered methylprednisolone at a dosage of 120 mg intravenously once daily, alongside lansoprazole at 30 mg intravenously once daily. By the 7th day of hospitalization, the serum amylase level had decreased to 432.00 U/L.

Adjustment for refractory pancreatitis: on the ninth day of admission, the patient reported exacerbated abdominal pain, which was diagnosed as steroid-refractory pancreatitis. Consequently, mycophenolate mofetil was introduced at a dosage of 1 g orally twice daily, in addition to the ongoing corticosteroid treatment. On the 10th day of admission, the methylprednisolone dosage was modified to 100 mg intravenously once daily. Subsequent laboratory tests indicated a serum amylase level of 466.70 U/L.

Further adjustments: on the 15th day of admission, the patient reported feeling generally weak and having a poor appetite. Lab tests showed a serum amylase level of 263.00 U/L. The methylprednisolone dose was lowered to 80 mg intravenously once a day. On the 16th day, the mycophenolate mofetil dose was reduced to 0.5 g orally twice a day, while maintaining the methylprednisolone at 80 mg intravenously once daily.

**Figure 3.**
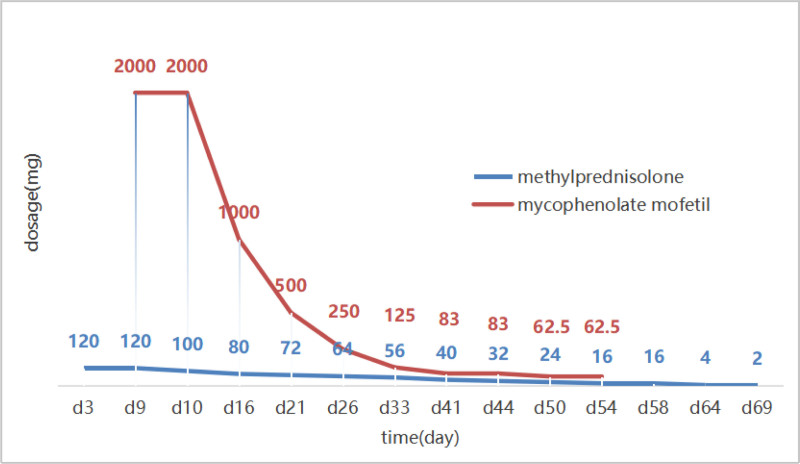
Dose adjustment of glucocorticoids and mycophenolate mofetil. On the third day of hospitalization, the patient was diagnosed with immune-mediated pancreatitis. Treatment with cadonilimab was immediately discontinued, and high-dose methylprednisolone was initiated. On the ninth day of hospitalization, the patient’s abdominal pain worsened, suggesting steroid-refractory pancreatitis. Mycophenolate mofetil was added to the glucocorticoid regimen, and the dose of methylprednisolone was gradually tapered. By the sixteenth day of hospitalization, the patient’s abdominal pain had improved, and the amylase levels had significantly decreased. The dose of mycophenolate mofetil was gradually reduced. Mycophenolate mofetil was tapered off over a period of 6 weeks, and glucocorticoids were tapered off over a period of 9 weeks. The 125 mg value represents the mean daily exposure of mycophenolate mofetil when given once every 2 days, 83 mg when given once every 3 days, and 62.5 mg when given once every 4 days. To depict the glucocorticoid-tapering trajectory, oral prednisone doses were uniformly converted to methylprednisolone equivalents (5 mg prednisone ≈ 4 mg methylprednisolone) and are presented throughout as methylprednisolone doses.

Improvement and tapering: By the 17th day, the patient’s appetite, general weakness, and abdominal pain had improved significantly, and the serum amylase level had decreased substantially. The immunosuppressive regimen was tapered according to a predefined schedule. Mycophenolate mofetil was discontinued stepwise over 6 weeks: 0.5 g twice daily for 4 days, then 0.25 g twice daily for 5 days, followed by 0.25 g once daily for 7 days, 0.25 g every other day for 7 days, 0.25 g every third day for 7 days, and finally 0.25 g every fourth day for 7 days before cessation. Glucocorticoids were weaned over 9 weeks: intravenous methylprednisolone was switched to oral prednisone, which was then reduced as follows—100 mg once daily for 2 days, then 90 mg, 80 mg, 70 mg, 60 mg, 50 mg, 40 mg, 30 mg, 20 mg, 10 mg, 5 mg once daily for 5 days each step, and finally to 2.5 mg once daily for the last 4 days, at which point the patient died. The specific treatment and tapering methods are illustrated in Figure 3.

### 2.6. Outcomes

Eight days following the initiation of mycophenolate mofetil, the patient exhibited a marked improvement in both abdominal pain and serum amylase levels. Nevertheless, due to the progression of the disease, the patient was unable to continue receiving antitumor therapy with cadonilimab and subsequently pursued traditional Chinese medicine as an alternative antitumor treatment. Unfortunately, 2 months later, the patient succumbed to respiratory distress.

## 3. Discussion

This case involves a patient with advanced squamous cell lung cancer who progressed after multiple lines of therapy and currently has no standard treatment options available. Phase Ib/II clinical trials have shown that cadonilimab combined with anlotinib achieves an objective response rate of 51.0% to 60.0% as first-line treatment in advanced non-small cell lung cancer (NSCLC) without epidermal growth factor receptor/anaplastic lymphoma kinase/ROS proto-oncogene 1 driver mutations.^[10]^ A multicenter retrospective observational study^[11]^ reported a disease control rate of 39.1% with cadonilimab in fourth-line or later treatment of advanced NSCLC. Thus, cadonilimab offers a therapeutic option for heavily pretreated NSCLC patients.

Immune checkpoint inhibitors (ICIs)-related pancreatic injury (PI) predominantly manifests as pancreatitis and elevated levels of lipase/amylase.^[12,13]^ A systematic review and meta-analysis^[14]^ of Phase III randomized controlled trials systematically assessed the incidence of immune-related pancreatitis, reporting an overall incidence of 0.81%, with a rate of grade 3 or higher at 0.45%. When 2 ICIs were administered in combination, the incidence of pancreatitis was observed to be 0.49%. Cadonilimab is a tetravalent bispecific IgG1 antibody that targets human PD-1 and CTLA-4, characterized by a symmetric IgG single-chain variable fragment structure. It is engineered with an Fc null configuration to abrogate antibody-dependent cell cytotoxicity, antibody-dependent cellular phagocytosis, complement-dependent cytotoxicity, and cytokine release. The design of cadonilimab is intended to eliminate Fc receptor binding and effector functions, thereby enhancing the efficacy and safety profile of the antibody drug.^[15]^ Patients undergoing treatment with this drug may experience immune-related adverse reactions. A comprehensive search of databases, including CNKI, Wanfang, VIP, and PubMed (up to April 2025), yielded no reports of immune-related pancreatitis associated with cadonilimab treatment. The epidemiological characteristics of immune-related pancreatitis potentially induced by cadonilimab, such as its association with gender, age, underlying conditions, timing of onset, clinical manifestations, and imaging features, remain undetermined.

At present, the primary manifestation of PI associated with ICIs induced by cadonilimab is the asymptomatic elevation of lipase levels. A comprehensive review of databases, including CNKI, Wanfang, VIP, and PubMed, identified 3 clinical trials^[16-18]^ investigating cadonilimab monotherapy for antitumor therapy. These trials encompassed a total of 316 patients, among whom 20 exhibited elevated lipase levels, corresponding to an incidence rate of 6.33%, with 4 patients experiencing elevations of grade 3 or higher, representing an incidence rate of 1.27%. Additionally, 7 patients demonstrated elevated amylase levels, with an incidence rate of 2.22%, and 1 patient presented with grade 3 or higher, yielding an incidence rate of 0.32%. Notably, no cases of pancreatitis adverse reactions were reported. In our institution, among 480 patients who received cadonilimab for antitumor therapy from April 2022 to December 2024, 1 patient exhibited elevated lipase levels, classified as grade 2 according to CTCAE version 5.0, with an incidence rate of 0.21%. Furthermore, 1 patient developed immune-related pancreatitis, classified as grade 3 according to CTCAE version 5.0, also with an incidence rate of 0.21%, which is lower than the incidence observed in the clinical trials.

The precise mechanism through which cadonilimab induces pancreatitis remains unclear.^[19]^ Research indicates a significant association between the co-inhibition of immune checkpoint receptors CTLA-4 and PD-1 and the onset of acute pancreatitis.^[20]^ CTLA-4 functions by inhibiting the proliferation and activity of T lymphocytes, primarily through the suppression of the phosphoinositide-3-kinase/protein kinase B signaling pathway, cyclin D3, cyclin-dependent kinases 4/6, and nuclear factor κB. The absence of CTLA-4-mediated negative immune regulation may reduce the activation threshold of lymphocytes, potentially facilitating the development of autoimmune diseases.^[21]^ Similarly, the PD-1/PD-L1 pathway plays a critical role in inhibiting T-cell activation and proliferation, promoting T-cell apoptosis, and facilitating evasion from host immune surveillance.^[22]^ This pathway is implicated in the immune regulation of acute pancreatitis via its effects on T lymphocytes.^[23]^ Consequently, based on the existing literature, it is hypothesized that cadonilimab may disrupt pancreatic immune homeostasis by inhibiting the CTLA-4 and PD-1/PD-L1 pathways, thereby contributing to PI.^[21]^ Owing to its unique molecular architecture, Fc-silenced design, and tumor-enriching properties, the mechanisms underlying cadonilimab-associated pancreatitis may differ from the combination regimen of CTLA-4 monoclonal antibody and PD-1 monoclonal antibody; however, no study has yet elucidated these differences, and in-depth investigations are urgently needed.^[24]^

Following 1 cycle of treatment with cadonilimab in conjunction with anlotinib, the patient exhibited elevated serum amylase levels and occasional abdominal pain. However, these symptoms were not deemed significant, and alternative etiologies could not be excluded, rendering the diagnosis of immune-related pancreatitis inconclusive. According to the National Comprehensive Cancer Network (NCCN) Guidelines for Management of Immunotherapy-Related Toxicities (2025.V1),^[25]^ hereafter referred to as the NCCN Guidelines, asymptomatic elevation of amylase does not necessitate the discontinuation of ICIs. Furthermore, literature suggests that decisions regarding the cessation of ICIs should be tailored to individual patient and disease characteristics, the severity of immune-related adverse events, and the therapeutic response.^[26]^ After thorough discussion between the clinical physician and the patient, a decision was made to monitor the condition while continuing treatment with cadonilimab and anlotinib. Following the second cycle of immunotherapy combined with targeted therapy, the patient returned to the local hospital for further investigation into the cause of the elevated amylase and to receive appropriate treatment, the details of which are unknown.

Upon the patient’s readmission for the third cycle of immunotherapy in conjunction with targeted therapy, an elevation in serum amylase levels was observed compared to the previous hospitalization. The patient continued to experience occasional abdominal pain, although the symptoms remained mild. Subsequent MRI findings indicated acute pancreatitis. Given the persistent elevation of serum amylase, ongoing abdominal discomfort, and radiological confirmation of acute pancreatitis, there was a strong suspicion of immune-related pancreatitis. According to the NCCN Guidelines,^[25]^ the severity of the immune-related pancreatitis in this patient was classified as moderate grade 3. It was recommended to temporarily discontinue immunotherapy and initiate intravenous fluid resuscitation. In cases where intravenous fluid resuscitation and pain management proved insufficient, treatment with prednisone or intravenous methylprednisolone at a dosage of 0.5 to 1 mg/kg/d was advised. The patient ceased treatment with cadonilimab. Due to the absence of detailed information regarding the patient’s treatment at the local hospital, it remains uncertain whether he received intravenous fluid resuscitation or corticosteroid therapy. Given the extended period of elevated amylase levels and persistent abdominal pain, the patient was administered high-dose methylprednisolone in accordance with the corticosteroid guidelines for severe grade 4 toxicity as outlined in the NCCN Guidelines.^[25]^ However, after 6 days of treatment, the patient’s abdominal pain intensified, leading to a suspicion of steroid-refractory pancreatitis. The NCCN Guidelines^[25]^ recommend that severe or steroid-refractory immune-related adverse events be managed with immunosuppressive agents. In accordance with the 2023 Guidelines from the Chinese Society of Clinical Oncology for the Management of Immune Checkpoint Inhibitor-Related Toxicities,^[27]^ patients experiencing grade 2 or higher immune-related pancreatitis who exhibit inadequate responses to corticosteroid therapy may consider combination therapy involving mycophenolate mofetil. There is no established standard for salvage therapy of steroid-refractory immune-mediated pancreatitis. Although isolated reports^[28-31]^ have described remission with infliximab, mycophenolate mofetil, azathioprine, or rituximab, head-to-head data comparing efficacy and safety among these immunomodulators are lacking. After weighing drug availability, administration convenience, and adverse event profiles in China, mycophenolate mofetil was selected as the additional immunomodulatory agent.

## 4. Conclusion

With the increasing utilization of ICIs, there is a potential rise in the incidence and severity of immune-related PI, which may adversely affect patients’ quality of life and treatment outcomes.^[32]^ Presently, immune-related PI associated with cadonilimab predominantly presents as an asymptomatic elevation of lipase levels, with immune-related pancreatitis being an infrequent adverse event. It is imperative for clinicians and clinical pharmacists to remain vigilant regarding this condition. Furthermore, this case represents a rare instance of successful management of steroid-refractory immune-related pancreatitis using mycophenolate mofetil, thereby offering valuable insights for the clinical management of similar cases.

## Acknowledgments

The authors would like to thank all the reviewers who participated in the review. Thanks to Kimi and Deepseek for their assistance in language translation and polishing.

## Author contributions

**Data curation:** Jin-lan Li.

**Resources:** Jin-lan Li.

**Supervision:** Jin-lan Li

**Writing – original draft:** Rui Qiu.

**Writing – review & editing:** Rui Qiu, Gao-feng Zheng, Min Zhong, Jin-lan Li.
